# Utility of Serum EB Virus Zta Antibody in the Diagnostic of Nasopharyngeal Carcinoma: Evidences From 2,126 Cases and 15,644 Controls

**DOI:** 10.3389/fonc.2019.01391

**Published:** 2019-12-19

**Authors:** Guangying Zhang, Zhanzhan Li, Qin Zhou

**Affiliations:** Department of Oncology, Xiangya Hospital, Central South University, Changsha, China

**Keywords:** nasopharyngeal carcinoma, EB virus, Zta antibody, diagnostic, meta-analysis

## Abstract

We collected previous published data and performed a systematical assessment for the diagnostic value of serum Zta antibody in NPC patients. Using bivariate-mixed effect model, we calculated the sensitivity, specificity, positive likelihood ratio (PLR), negative likelihood ratio (NLR), diagnosis odds ratio (DOR), and summary receiver operating characteristics curve (AUC) and their 95% confidence intervals (CIs). We also performed subgroup analysis to explore the heterogeneity. We included 23 studies including 24 pieces of data and 17,770 study subjects (2,126 cases and 15,644 controls). The overall combined sensitivity was 0.85 (95%CI: 0.80–0.89) and the combined specificity was 0.90 (95%CI: 0.87–0.93). The summarized AUC was 0.94 with 95%CI of 0.92–0.96. The PLR was 8.9 (95%CI: 6.4–12.2) and the NLR was 0.17(95%CI: 0.12–0.23). The diagnostic odds ratio was 53 (95%CI: 32–87). For publication year, the sensitivity was 0.88 (95%CI: 0.84–0.91) and the specificity was 0.90 (95%CI: 0.84–0.93) for ≤2006. The AUC, PLR, NLR and DOR were 0.94, 8.8, 0.13, and 64. The pooled results were similar for >2006 group. For different sample size, the pooled AUC was 0.94 for ≤Median and was 0.95 for >Median that were very close to the overall estimations. For different population setting, no overlap was found in the sensitivity (0.84 vs. 0.87), specificity (0.90 vs. 0.84), PLR (8.7 vs. 5.5), NLR (0.16 vs. 0.08–0.33), DOR (49 vs. 35), and AUC (0.94 vs. 0.92) between Asian and others. The serum EBV antibody examination has high diagnostic accuracy for early-stage NPC. The diagnostic accuracy seems not to be influenced by sample size, publication year, and ethnic. Considering the few numbers of study with non-Asian population, the present results need to be confirmed in other population setting.

## Introduction

Nasopharyngeal carcinoma (NPC) is located in the nasopharyngeal mucosa and belongs to one of malignant tumor of head and neck (1). NPC is geographically distributed in certain population. Worldwide, NPC is mainly distributed in Southeast Asia, Alaska Eskimos, Greenland, Central Africa, and North Africa. The south of China is one of high-incidence areas (2). NPC has different stage. Although, in the past decades, the survival status of NPC patients has been greatly improved with the development of radiotherapy technical and other therapy methods (3). The 5-years overall survival rate was more than 90% in the early stage of diseases. However, the 5-years overall survival rate was only about 50% in the patients with advanced stage (4). The improvement of long-term survival status is very limited (5). Due to the cryptic onset, NPC is usually presented with a loco-regionally advanced state at diagnosis. Therefore, critical importance lies in developing more sensitive and specific test for the screening and early diagnosis.

Epstein-Barr virus (EBV) is correlated with etiology and pathogenesis of NPC in endemic regions (6). The anti-EBV antibody level is significantly elevated. The enzyme-linked immunosorbent assay (ELISA) based on recombinant EBV is taking the place of traditional serologic markers, such as VCA IgA and EA IgA (7). Zta protein, also called Zebra, EB1, or Z protein, is encoded by EBV BZLF1 gene. In NPC patients, the EBV cautiously expressed (EB nuclear antigen 1) EBNA 1 and Zta encoded by BZLF1 were the key enzymes that can regulate EBV from to incubation period to replicative period (8, 9). It was also reported that ZEBRA/IgG antibody was detected in the serum of NPC patients and can be a marker of early-diagnosis and prognosis assessment (10). A lot of studies have assessed the clinical utility of Zeta antibody in the diagnosis of NPC patients (10–12). However, the sensitivity and specificity were different due to different sample size, ethnic and others factors. A comprehensive evaluation is required for such an index. In the present, we collected previous published data and performed a systematical assessment for the diagnostic value of serum Zta antibody in NPC patients.

## Materials and Methods

This is a meta-analysis based on previous studies. The ethnic approval was not required.

### Search Strategy

We performed an online search for relevant studies using the following databases: PubMed, China National Knowledge Infrastructure, Web of Science, Embase, Wanfang, and Google scholar. The search date is updated to November 15th, 2019. We placed language restrictions in Chines and English. The following keywords were employed for literature retrieval: (“nasopharyngeal carcinoma” OR “NPC” OR “nasopharynx cancer”; “EB virus OR EBV”, “ZEBRA OR Zta,” “diagnoses” OR “diagnostic value” OR “sensitivity and specificity” OR “ROC curve” OR “receiver” operating characteristics. We also reviewed the references lists of relevant studies for potential eligible studies. The latest data was used for the replicated data. We performed this meta-analysis by following the Preferred Reporting Items for Systematic review and Meta-Analysis checklist (Supplementary Material 1). The search strategy was presented in the Supplementary Material 2.

### Study Selection and Data Extraction

The included must meet the following criteria: (1) studies assessing the diagnostic of serum EB virus Zta antibody in the NPC; (2) studies with sufficient data for analysis, including true positive (TP), false positive (FP), false negative (FN), and true negative (TN); (3) NPC was confirmed by pathology examination; (4) for the same data, the latest results were used. The following studies were excluded: (1) studies without sufficient data were excluded. (2) Study was settled in a specific population. (3) Reviews, letters, comments, editorials, and case reports were also excluded.

Two investigators interpedently extracted the data and the disagreements were solved by consensus. For each study, the following data was extracted: the surname of the first author, publication year, ethnicity, sample size including case and control, examination methods, gold standard, 4-folds data (TP, FP, FN, TN), and sensitivity and specificity of each study.

### Quality Assessment

We used the Quality Assessment of Diagnostic Accuracy Studies (QUADAS-2) to assess the quality of included study (13). This tool has been widely used in the diagnostic accuracy studies. The QUADAS tool assessed the study quality from two domains: risk of bias and concerns about application. The risk of bias consists of four sub-items: patient selection, index test, reference standard, and flow of patients through the study and timing of the index tests and reference standard. Risk of bias is considered as “high” “unclear” or “low.” If the answers to all sub-items for a domain are “yes,” then risk of bias can be treated as low. If any of sub-item is judged “no,” potential bias may exist. Concerns about applicability are scored as “high” “unclear” or “low.”

### Statistical Analysis

First, we assessed the threshold effect using the Pearson coefficient (*r* = 0–0.341, *P* = 0.103). The threshold effect determined which model was used (14). No threshold effect existed for the present study. And the bivariate mixed effects model was used. We calculated the following parameters and their 95% confidence internals (CIs): sensitivity, specificity, positive likelihood ratio (PLR), negative likelihood ratio (NLR), diagnosis odds ratio (DOR), and summary receiver operating characteristics curve (AUC), An AUC of 1.0 represents the perfect discrimination ability (15–17). The heterogeneity within studies was examined using Q test and I^2^ statistic. *P* < 0.05 and I^2^ > 50% indicated the significant heterogeneity (18, 19). Fagan' plot and the line graph of post-test probabilities vs. prior probabilities between 0 and 1 using summary likelihood ratios (20). Sensitivity analysis: quantile plot of residual-based goodness-of fit and Chi-squared probability plot of squared Mahala Nobis distances were used for assessment of the bivariate normality assumption; spike plot was used for checking for particularly influential observations using Cook's distance. Scatterplot was used for checking for outliers using standardized predicted random effects. The publication bias was assessed by Deek's funnel plot asymmetry test (21). No overlap between two confidence intervals indicated significant difference. All analyses were completed on Stata 14.0 and Reviewer manager 5.0. *P* < 0.05 was considered as significant level.

## Results

### Study Selection and General Characteristics

**Figure 1:**
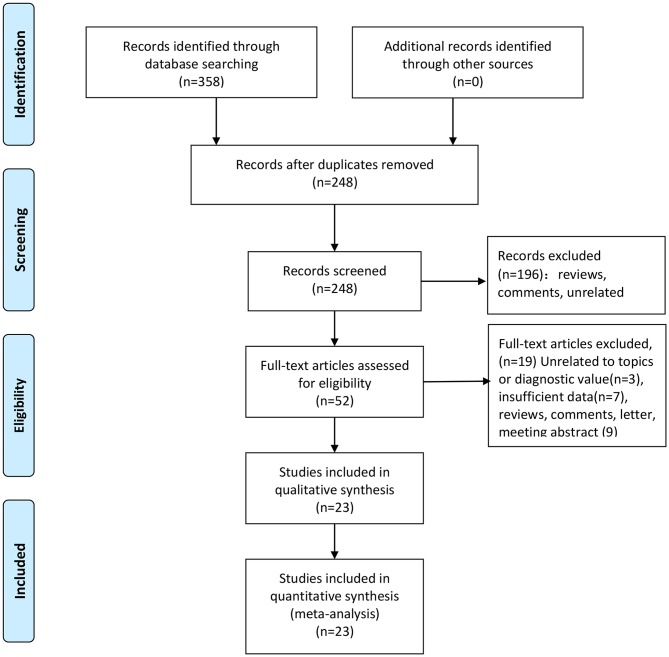
Flow chart of literature selection.

We totally obtained 358 articles from six online electronic database. 110 articles were excluded because of duplicates data and publications. We checked the titles and abstracts of 248 articles and removed 196 articles because they are significantly unrelated topics and others publications, such as reviews and comments. We downloaded the full-text of 52 articles for further screening. Among of these articles, seven studies with insufficient data, three articles with unrelated topics or diagnostic values, and nine articles belonged to reviews, comments, letter and meeting abstract. At last, we included 23 studies including 24 pieces of data (Supplementary Material 3). The selection flow of study selection is presented in Figure 1. The total sample size is 17,770 with 2,126 cases and 15,644 controls. These studies were published from 2003 to 2018. All cases were confirmed by pathology examination. The examination of antibody was ELISA. The highest sensitivity was 0.96 and the lowest was 0.36. The highest specificity was 0.97 and the lowest was 0.81. The distributions of 4-folds (TP, FP, TN, FN) and details were shown in Table 1.

**Table 1:** General characteristics of included study in the meta-analysis.

**Author**	**Year**	**Examination**	**Gold standard**	**Case**	**Control**	**TP**	**FP**	**FN**	**TN**	**Sensitivity**	**Specificity**
Gu	2003	ELISA	Pathology	57	58	46	11	11	47	0.81	0.81
Hu	2006	ELISA	Pathology	85	132	75	16	10	116	0.88	0.88
Liang	2008	ELISA	Pathology	195	188	163	31	32	157	0.84	0.84
Zhang1	2006	ELISA	Pathology	288	96	262	5	26	91	0.91	0.95
Zhang2	2006	ELISA	Pathology	7	7,473	5	292	2	7,181	0.71	0.96
Ren	2006	ELISA	Pathology	59	59	53	6	6	53	0.90	0.90
Jiang	2009	ELISA	Pathology	49	89	33	9	16	80	0.67	0.90
Yi	2007	ELISA	Pathology	24	28	22	3	2	25	0.92	0.89
Cheng1	2007	ELISA	Pathology	41	90	36	3	5	87	0.88	0.97
Cheng2	2003	ELISA	Pathology	85	256	69	41	16	215	0.81	0.84
Dardari	2000	ELISA	Pathology	178	180	168	5	10	175	0.94	0.97
Joab	1991	ELISA	Pathology	41	200	36	50	5	150	0.88	0.75
Li	1994	ELISA	Pathology	23	98	23	3	0	95	0.96	0.97
Cao	1998	ELISA	Pathology	77	30	70	0	7	30	0.91	0.97
Cheng	2002	ELISA	Pathology	121	332	96	66	25	266	0.79	0.80
Zheng	2006	ELISA	Pathology	232	602	215	39	17	563	0.93	0.94
Chan	2003	ELISA	Pathology	55	163	41	28	14	135	0.75	0.83
Zeng	1992	ELISA	Pathology	28	103	24	37	4	66	0.86	0.64
Chen	2018	ELISA	Pathology	100	500	90	50	10	450	0.90	0.90
Gu	2016	ELISA	Pathology	60	60	53	2	7	58	0.88	0.97
Yu	2016	ELISA	Pathology	152	675	91	35	61	640	0.60	0.95
Zhang3	2015	ELISA	Pathology	113	228	41	8	72	220	0.36	0.96
Hu	2014	ELISA	Pathology	36	3,004	29	601	7	2,403	0.81	0.90
Wang	2011	ELISA	Pathology	20	1,000	18	167	2	833	0.90	0.83

*TP, true positive; FP, false positive; FN, false negative; TN, true negative; ELISA, enzyme-linked immunosorbent assay*.

### Quality Assessment

**Figure 2:**
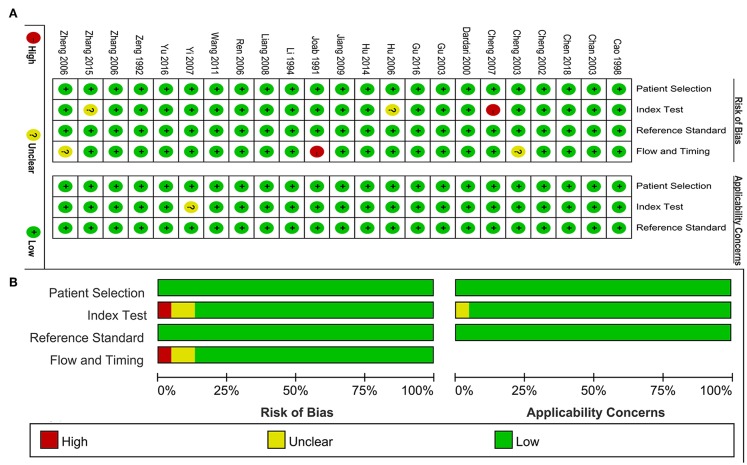
Quality assessment of included study. **(A)** Review authors' judgements about each domain presented as percentages across included studies. **(B)** Review authors' judgements about each domain for each included study.

The quality assessment of included studies was summarized in the Figures 2A,B. Figure 2A presented the risk of bias and applicability concerns of each study. One study was judged as high-risk bias because of the flow and timing issue. One study was considered as high-risk bias because of the index test. The ratio of studies with high-risk bias was 8.7%. Two studies were judged as unclear risk bias in the item of flow and timing. Two studies had unclear risk bias in the item of index test. One study was scored as unclear applicability concern in the index test. The ratio of unclear risk studies was 17.4%. The ratio of studies with unclear concerns regarding applicability was 4.3%. On the whole, the included studies achieved a high point in the quality assessment.

### Pooling Results

**Figure 3:**
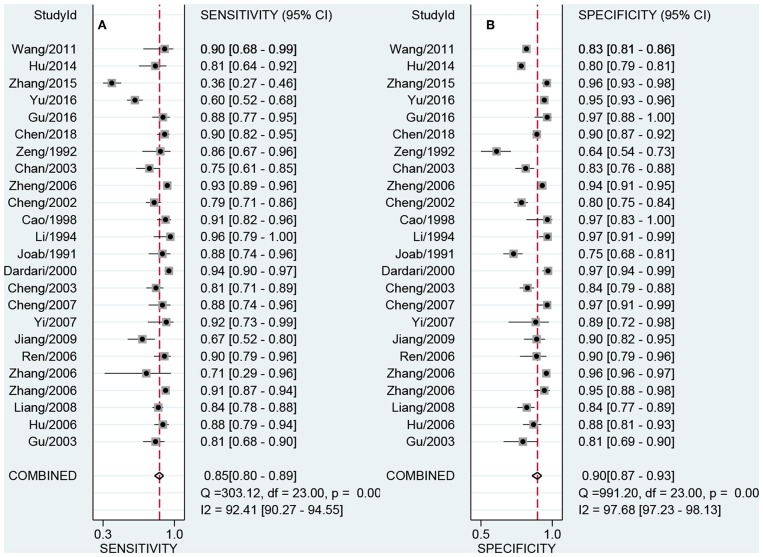
Forest plot of pooled sensitivity **(A)** and specificity **(B)**.

**Figure 4:**
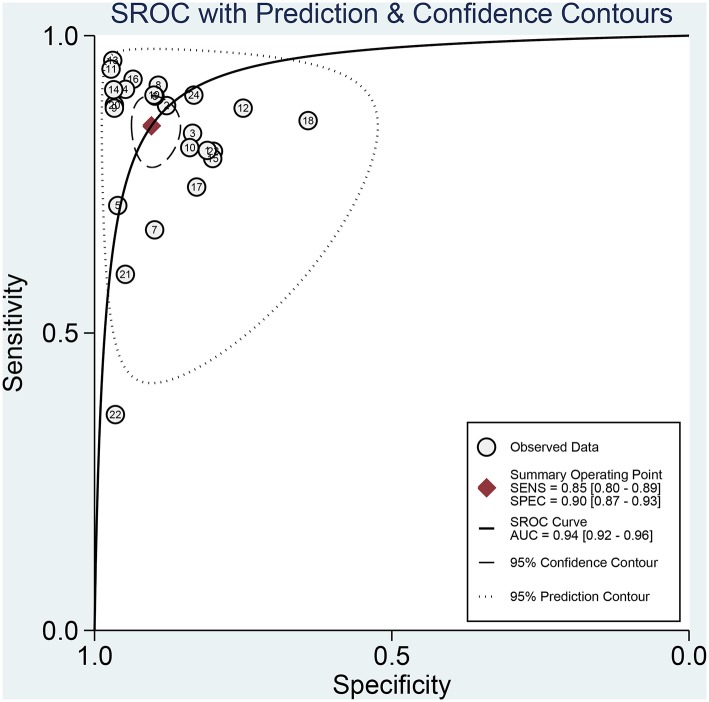
The SROC curve of serum Zta antibody for NPC.

**Figure 5:**
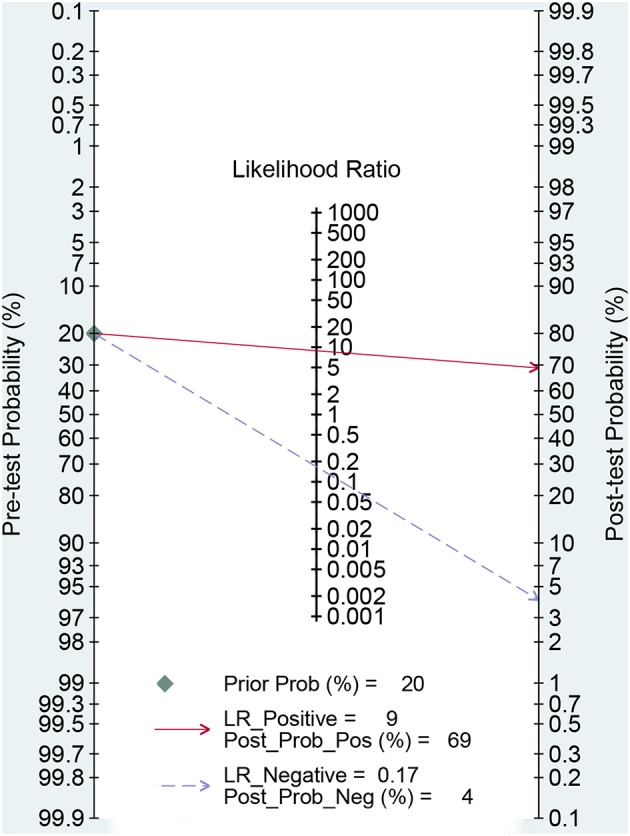
Fagan diagram assessing the overall diagnostic value of serum Zta antibody for NPC.

Figures 3A,B presented the combined results of sensitivity and specificity. The overall combined sensitivity was 0.85 (95%CI: 0.80–0.89) and the combined specificity was 0.90 (95%CI: 0.87–0.93) that were from the random-effect model. Because the heterogeneity was high (*P* < 0.05 and I^2^ > 50%). The summarized AUC was 0.94 with 95%CI of 0.92–0.96 (Figure 4). The PLR was 8.9 (95%CI: 6.4–12.2) and the NLR was 0.17 (95%CI: 0.12–0.23). The diagnostic odds ratio was 53 (95%CI: 32–87). According to the criteria, PLR > 10 and NLR < 0.1 indicated high accuracy. According the diagnostic criteria, the EBV Zta antibody examination achieved a high diagnostic ability for NPC. The Figure 5 shows the pre-test probability and post-test probability. Based on the PLR, the post-test probability could arrival at 69%.

Because the heterogeneity within studies is high. We also performed subgroup analysis in the publication year (Median ≤2006 vs. >2006), sample size (≤Median vs. >Median) and ethnic (Asian vs. others). For publication year, the sensitivity was 0.88 (95%CI: 0.84–0.91) and the specificity was 0.90 (95%CI: 0.84–0.93) for ≤2006. The AUC, PLR, NLR and DOR were 0.94, 8.8, 0.13, and 64. The pooled results were similar for >2006 group. The values of six parameters were 0.80, 0.91,0.93, 8.8, 0.22, and 40. For different sample size, the pooled AUC was 0.94 for ≤Median and was 0.95 for >Median that were very close to the overall estimations. For different population setting, no overlap was found in the sensitivity (0.84 vs. 0.87), specificity (0.90 vs. 0.84), PLR (8.7 vs. 5.5), NLR (0.16 vs. 0.08–0.33), DOR (49 vs. 35), and AUC (0.94 vs. 0.92) between Asian and others. The details of results were presented in the Table 2.

**Table 2:** Summary estimated of diagnostic performance of high *b*-value diffusion weighted Imaging for prostate cancer detection.

**Category**	**SEN (95%CI)**	**SPE (95%CI)**	**PLR (95%CI)**	**NLR (95%CI)**	**DOR (95%CI)**	**AUC (95%CI)**
**Overall**	0.85 (0.80–0.89)	0.90 (0.87–0.93)	8.9 (6.4–12.2)	0.17 (0.12–0.23)	53 (32–87)	0.94 (0.92–0.96)
**Publication year**						
≤2006	0.88 (0.84–0.91)	0.90 (0.84–0.93)	8.6 (5.4–13.8)	0.13 (0.10–0.19)	64 (30–137)	0.94 (0.92–0.96)
>2006	0.80 (0.69–0.88)	0.91 (0.86–0.94)	8.8 (6.1–12.6)	0.22 (0.14–0.35)	40 (23–70)	0.93 (0.91–0.95)
**Sample size**						
≤Median	0.84 (0.76–0.90)	0.90 (0.85–0.93)	8.2 (5.4–12.6)	0.18 (0.12–0.27)	46 (23–90)	0.94 (0.91–0.95)
>Median	0.86 (0.80–0.91)	0.91 (0.86–0.94)	9.7 (6.1–15.2)	0.15 (0.10–0.22)	65 (32–129)	0.95 (0.92–0.96)
**Ethnic**						
Asian	0.84 (0.78–0.88)	0.90 (0.87–0.93)	8.7 (6.3–11.9)	0.18 (0.13–0.24)	49 (30–79)	0.94 (0.91–0.96)
Others	0.87 (0.76–0.93)	0.84 (0.64–0.94)	5.5 (2.1–14.8)	0.16 (0.08–0.33)	35 (7–178)	0.92 (0.89–0.94)
**Sensitivity analysis**						
Excluding study 22	0.86 (0.82–0.89)	0.90 (0.86–0.93)	8.7 (6.2–12.1)	0.15 (0.12–0.20)	56 (34–94)	0.94 (0.91–0.96)
Excluding study 18	0.85 (0.80–0.89)	0.91 (0.88–0.93)	9.4 (6.9–12.8)	0.17 (0.12–0.23)	57 (34–93)	0.94 (0.92–0.96)
Excluding study 11	0.84 (0.79–0.88)	0.90 (0.86–0.93)	8.3 (6.1–11.3)	0.18 (0.13–0.24)	47 (29–74)	0.94 (0.91–0.95)

*SEN, sensitivity; SPE, specificity; PLR, positive likelihood ratio; NLR, negative likelihood ratio; DOR, diagnostic odds ratio; AUC, area under the curve*.

### Sensitivity Analysis and Publication Bias

**Figure 6:**
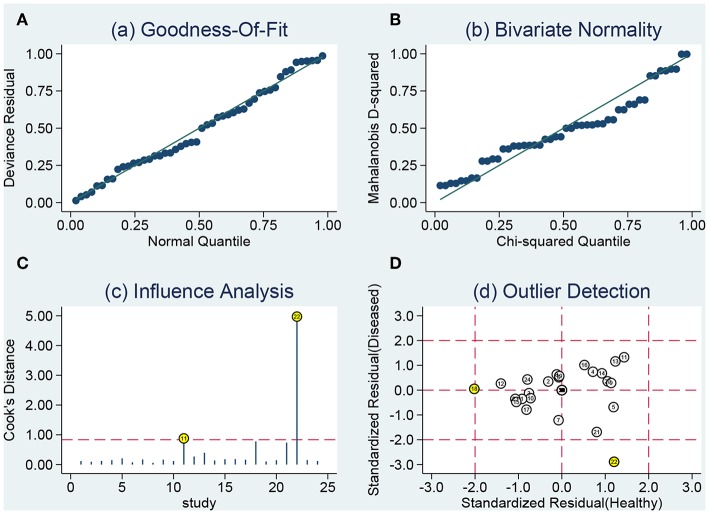
Sensitivity analyses: graphical depiction of residual based goodness-of-fit **(A)**, bivariate normality **(B)**, influence **(C)**, and outlier detection **(D)** analyses.

**Figure 7:**
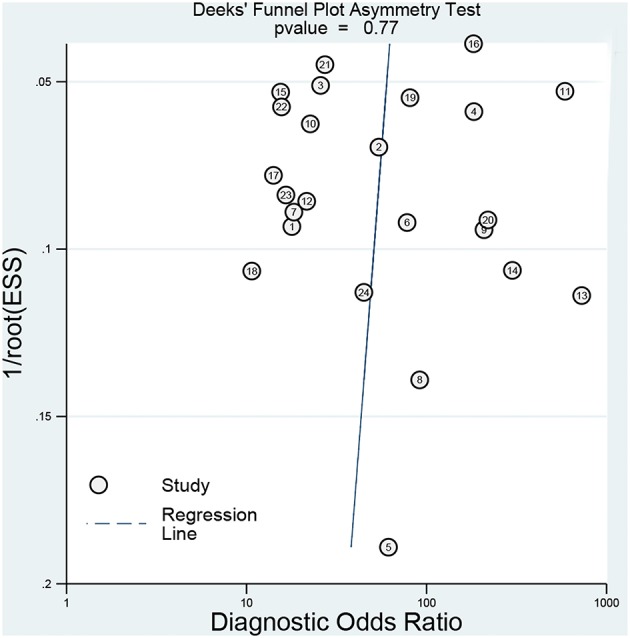
Deeks' plot for the assessment of publication bias (the closer to 90° the regression line is, the less significant the publication bias is).

The sensitivity analysis was presented the Figure 6. The Goodness of fit plot (Figure 6A) and bivariate normality plot (Figure 6B) indicted studies distributed along the references line. The influence analysis (Figure 6C) suggested that only one study was beyond the reference line. The outlier detection (Figure 6D) indicated the same study the same as the influence analysis. Two study were out of the detection and the further pooled results found no significant change. All signs indicated the pooled results are stable and few studies can affect the pooling results. The Deeks's funnel plot (Figure 7) asymmetry test indicated no significance (*P* = 0.770), which means no publication bias exits.

## Discussion

Our results from a large sample size indicated that serum EBV Zta antibody can provide a high diagnostic accuracy with the sensitivity of 0.85 and the specificity of 0.90. In the diagnostic study assessment, PLR > 10 and NLR < 0.01 means the high diagnostic accuracy. Our pooping PLR and NLR were 8.9 and 0.17, achieving a moderate diagnostic accuracy. The higher the DOR is, the better diagnostic ability is. The calculated DOR was 64, suggesting the overall accuracy was high. While the AUC was 0.94, close to 1, indicating the high diagnostic accuracy. All results indicated serum Zta antibody is enough for early-stage screening.

For significant different prognosis in early stage and advanced stage NPC patients, it is in great need of effective and convenient diagnostic methods. The serological diagnosis has become an important selection for early-stage screening of NPC. The EBV antibody spectrum can be detected when people get infected with EBV (22). Furthermore, the serum EBV antibody presented continuous elevated levels before routine symptoms came out in patients (23). This condition particularly increased the risk of NPC. The antibody detected by serology is mainly EBV-related antibody, including EBV capsid antigen (VCA), early intracellular antigen (EA), EB virus associated nuclear antigen (EBNA), and EB virus immediate early proteins (Zta and Rta) (24). The EB VCA antibody is a kind of structural protein that is synthesized during proliferation end stage. The VCA is also the most widely studied and applicated EBV antigen in clinical practice (25). However, the specificity was very poor because the high positive rate was also found in normal health population. The EBV EA is marker presenting virus activity proliferation, including dispersed type and localized type. It was reported that serum EA antibody was mainly combined with disperse type antibody and mediated specific responses (26). The high specificity can be achieved when IgA antibody was detected. However, the sensitivity was very poor, which means patients could be ignored during diagnostic period. The EBNA1 antigen was widely expressed in all cells with EBV genes, mainly played an important role in the latent infections of EBV, was associated with EBV gene copy and stability, and was necessary for cell translation (27). Moreover, the positive rate was the easiest high. Therefore, this protein can be a biomarker of NPC detection. The BZLF1 transcription activator (Zta) is encoded by immediate early genes, namely BALF1. Previous study reported that Zta was a key molecular promoting latent period to proliferation period. While Rta was similar to Zta and have a synergistic effect with Zta. Both of Zta and Rta can activate the EBV proliferation (28). The combined detection will help an early-stage confirm of NPC using the enzyme linked immunosorbent assay. The present results confirmed that Even solo Zta antibody examination can achieved a good accuracy. Of course, the EBV DNA examination from real-time quantitative polymerase chain reaction had higher sensitivity and specificity and antibody examination from ELISA methods (29). The EBV DNA copy number not only provided better judgment for diagnostic and clinical stage but also used for NPC local recurrence and distant metastasis (30). However, the EBV copy examination had higher positive rate in high-risk population and the utility for early-stage screening and NPC progression is very limited.

The reason why the Zta can be a key protein of EBV infection proliferation is associated with itself structure. Zta has an important structure of its own positive feedback activation, which can bind to the ZREs site at the starting point of viral genome cleavage replication and recruit the core replication protein to the starting point of replication. Meanwhile, the core replication protein can also promote the preferential binding of Zta protein to Ori-Lyt (31). This process promotes virus replications. It was also thought that It has also been suggested that weakened DNA methylation of EB virus genome during cleavage reduces the binding of Zta to CpGZREs, which may be the regulatory switch of gene initiation during cleavage (32). However, this does not affect the interaction between other sites of Zta and abundant non-cpg ZREs, resulting in the continuous binding of Zta to reaction sites. Besides, interaction can occur between Zta and P53 and CBP that created an advantage condition for virus replicating (33).

The present study has some limitations. Firstly, NPC is a geographically distributed disease. The included studies of this meta-analysis are mainly from Asian population. The present results need to be confirmed in other population setting. Second, study population of each study included different stage NPC patients. The specific data for different stage is unavailable. Third, the EBV can also cause other diseases. The antibody detection can also cause some false positive cases. Finally, previous study reported that antibody combined examination can improve the sensitivity and specificity (34). Besides, the heterogeneity is high within studies. we performed the subgroup analysis in different publication year, sample size, and ethnic and did not find significant heterogeneity change. More data is required for heterogeneity. The future studies should screen which antibody combination can achieve the best diagnostic accuracy.

In conclusion, the serum EBV antibody examination has high diagnostic accuracy for early-stage NPC. The diagnostic accuracy seems not to be influenced by sample size, publication year, and ethnic. Considering the few numbers of study with non-Asian population, the present results need to be confirmed in other population setting. Meanwhile, the combination antibody examination should be performed for better diagnostic accuracy.

## Data Availability Statement

All datasets for this study are included in the article/Supplementary Material.

## Author Contributions

QZ designed the study. ZL performed the data analysis. GZ wrote the manuscript. All authors have read and approved the final version of the manuscript.

### Conflict of Interest

The authors declare that the research was conducted in the absence of any commercial or financial relationships that could be construed as a potential conflict of interest.

## Supplementary Material

Supplementary Material 1 PRISMA (Preferred Reporting Items for Systematic review and Meta-Analysis) checklist.

Supplementary Material 2 Search strategy in the database.

Supplementary Material 3 References lists of included in the meta-analysis.
