# Supplementary material for: Utility of Serum EB Virus Zta Antibody in the Diagnostic of Nasopharyngeal Carcinoma: Evidences From 2,126 Cases and 15,644 Controls
Source: Front Oncol. 2019 Dec 19;9:1391. doi: 10.3389/fonc.2019.01391 (PMC6930900; doi:10.3389/fonc.2019.01391)
Supplement: Supplementary Material 2 — Search strategy in the database. [file Data_Sheet_2.docx]

**Supplementary material 2** Search strategy in the database

1. **Search strategy in the English database**

Step 1. nasopharyngeal carcinoma [MeSH] OR NPC[ MeSH] OR nasopharynx cancer [ MeSH]

Step 2. EB virus [MeSH] OR EBV [MeSH]

Step 3. ZEBRA[ MeSH] OR Zta [ MeSH]

Step 4. diagnoses [MeSH] OR diagnostic value [MeSH] OR sensitivity MeSH] OR specificity [MeSH] OR ROC [MeSH] OR receiver operating characterisitcs

Step 5: Step 1 AND Step 2 AND Step 3 AND Step 4

Step 6 humans [MeSH] not animals [MeSH]

Step 7: Step 1 AND Step 2 AND Step 3 AND Step 4 not Step 6.

1. **Search strategy in the Chinese database**

**Step 1** 鼻咽癌 OR 鼻咽肿瘤

**Step 2** EB病毒 OR EBV

**Step 3** Zebra OR Zta OR IgG抗体

**Step 4** 诊断试验 OR 筛检 OR 灵敏度 OR 特异度 OR ROC OR 受试者工作特征曲线

**Step 5** Step 1 AND Step 2AND Step 3 AND Step 4
