# Supplementary material for: Utility of Serum EB Virus Zta Antibody in the Diagnostic of Nasopharyngeal Carcinoma: Evidences From 2,126 Cases and 15,644 Controls
Source: Front Oncol. 2019 Dec 19;9:1391. doi: 10.3389/fonc.2019.01391 (PMC6930900; doi:10.3389/fonc.2019.01391)
Supplement: Supplementary Material 3 — References lists of included in the meta-analysis. [file Data_Sheet_3.docx]

**Supplementary material 3** References lists of included in the meta-analysis(1-23)

**References:**

1. Chen SC, Wu JE, Zhang XJ, Chen D, Hu JY, Peng L. Application valus of VSA-IgA, NA1-IgA and Zta-IgA detection in screening of nasopharyngeal carcinoma. *LLab Med Clin*. (2018) 15: 1883-1884.

2. Gu XM, Li YY, Lv RR, Huang JS, Zhou XJ, Du GY. The correlation between serum homocysteine and EB virus three antibodies in nasopharyngeal carcinoma and the evaluation of its diagnosis performance. *Chin J Health Lab Tec*. (2016) 26: 231-233.

3. Yu X, Ji MF, Cheng WM, Huang YL, Li FK. Assessment of EBV antibodies and EBV-DNA in the disgnosis and stage of nasopharyngeal carcinoma. *Chin J Oncol*. (2016) 43: 650-654.

4. Zhang QF, Zhang XL, Ling GH, Zhou JH, Cao YP. Analysis of diagnositc value of EB virus antibody detectioin in the diagnosis of nasopharyngeal carcinoma. *Modern Med*. (2015) 13: 57-59.

5. Hu DF, Ling HY, Miao Y, Liang LL, Zhong Y, Bao AZ. The investigation of EB virus infection of Miao Buyi and Han population and evaluation of the diagnosis of Zta-lgA and VCA-IgA antibody for nasopharyngeal carcinoma in Guizhou. *Guizhou Med*. (2014) 38: 304-307.

6. Wang X, Zhao SP, Wu X, Zheng DZ. The Application of the detection of four protein antibody markers in screening and diagnosis of nasopharyngeal carcinoma. *Lab Med Clin*. (2011) 8: 2563-2564. doi:10.3969/j.issn.1672-9455.2011.21.002

7. Jiang SQ, Liu Q. Application of Logistic regression in combination with multiple diagnositc tests for auxiliary diagnosis of nasopharyngeal carcinoma. *Cancer (China)*. (2009) 28: 213-216.

8. Dardari R, Menezes J, Drouet E, Joab I, Benider A, Bakkali H, et al. Analyses of the prognostic significance of the Epstein-Barr virus transactivator ZEBRA protein and diagnostic value of its two synthetic peptides in nasopharyngeal carcinoma. *J Clin Virol*. (2008) 41: 96-103. doi:10.1016/j.jcv.2007.09.010

9. Liang YJ, Zong YS, Gu YL, Zhang Y, Feng YF, Liu YD, et al. Application of enzyme-linked immunosorbent assay to the serological diagnosis of nasopharyngeal carcinoma. *J Prac Med*. (2008) 24: 3055-3058. doi:10.3969/j.issn.1006-5725.2008.17.063

10. Yi X, Wu YY, Xie Y, Kang M, Tang AZ. Antibody response to recombinant Epstein-Barr virus Zta protein in patients with Nasopharyngeal carcinoma. *Guanxi Med Univ J*. (2007) 24: 365-367.

11. Cheng WM, Ji MF, Li XL, Su NH, Yang JH. Analysis of serum levels of antibodies against EBV EBNA1 and EBZta in individuals in a nasopharyngeal carcinoma clan who have non-nasopharyngeal carcinoma. *Chin Tumor Clin*. (2007) 34: 1238-1240.

12. Hu WW, Zong YS, Li FP, Li GM, Zhong BL, Zhang M, et al. Comparison of 6 antibody assays detecting Epstein-Barr virus for serodiagnosis of naropharyngeal carcinoma. *Chin J Oncol*. (2006) 33: 795-798.

13. Zhang XM, Zhong JM, Tang NZ, Zhang XG, Liao J, Zheng YM, et al. Comparison of IgA/VCA, IgA/EA, IgG/EA in immunoenzyme methods and ZEBRA ELISA in early diagnosis of nasopharyngeal carcinoma. *Chin J Exper clin Vriology*. (2006) 20: 263-265. doi:10.3760/cma.j.issn.1003-9279.2006.03.020

14. Tang MZ, Li J, Cai YL, Cheng JK, Mo YK. Detection of EB virus ZEBRA/IgG antibody in nasopharyngeal carcinoma serum diagnosis. *Acta Med Sinica*. (2006) 19: 1092-1093. doi:10.3969/j.issn.1008-2409.2006.06.016

15. Ren J, Zhang XM, Zhang XG, Li HX, Zhou L, Zeng Y. Studies on antibody response to recombinant Rta protein in patient with nasopharyngeal carcinoma. *Chin J Micro&Immu*. (2006) 26: 1057-1059. doi:10.3760/j:issn:0254-5101.2006.11.023

16. Cheng WM, Ji MF, Li XL, Yang JL, Zong YS. Screening out nasopharyngeal carcinoma by two-stage ELLSA for EB virus. *Chin J Immun*. (2003) 19: 834-836.

17. Chan KH, Gu YL, Ng F, Ng PS, Seto WH, Sham JS, et al. EBV specific antibody-based and DNA-based assays in serologic diagnosis of nasopharyngeal carcinoma. *Int J Cancer*. (2003) 105: 706-709. doi:10.1002/ijc.11130

18. Gu YL, Zhang CQ, Ng SP, Zong YS, Liang YJ, Chen YL. Study on sero-diagnosis of nasopharyngeal carcinoma using a dual antibody test against recombinant Epstein-Barr virus antigens. *Chin J Cancer*. (2003) 22: 903-906.

19. Cheng WM, Chen GX, Chen HL, Luo RX, Wu ZB, Lu YS, et al. Assessment of nasopharyngeal carcinoma risk by EB virus antibody profile. *Chin J Oncol*. (2002) 24: 561-563. doi:10.3760/j.issn:0253-3766.2002.06.013

20. Huang SM, Huang TB, Jian SW. The singnificance of EB virus ZEBRA/IgG in early diagnosis and porgnosis of nasopharyngeal carcinoma. *Cancer(China)*. (1998) 17: 4-8.

21. Li T, Zeng Y. Detection of IgG/Zebra antibodies in sera from patients with nasopharyngeal carcinoma by ELLSA methods. *Chin J Virology*. (1994): 78-80.

22. Zeng Y, Nicolas JC, G S. Detection of ZEBRA antibody in sera from patients with nasopharyngeal carcinoma infectious mononucleosis and burkitt lymphoma. *Bingdu Xuebao*. (1992) 8: 218-222.

23. Joab I, Nicolas JC, Schwaab G, De-The G, Clausse B, Perricaudet M, et al. Detection of anti-Epstein-Barr-virus transactivator (ZEBRA) antibodies in sera from patients with nasopharyngeal carcinoma. *Int J Cancer*. (1991) 48: 647-649.
